# Age-specific patterns of all-cause mortality across COVID-19 booster dose groups in two Japanese municipalities: an exploratory analysis

**DOI:** 10.3389/fpubh.2026.1795437

**Published:** 2026-04-20

**Authors:** Yasushi Iwamoto, Hiroshi Kusunoki, Yukari Kamijima, Ryota Sakai, Yumi Watanabe, Hideki Kakeya

**Affiliations:** 1 Graduate School of Economics, The University of Tokyo, Tokyo, Japan; 2 Department of Internal Medicine, Osaka Dental University, Hirakata, Japan; 3 Faculty of Pharmaceutical Sciences, Tokyo University of Science, Tokyo, Japan; 4 Department of Rheumatology and Clinical Immunology, Saitama Medical Center, Saitama Medical University, Kawagoe, Japan; 5 Division of Preventive Medicine, Niigata University Graduate School of Medical and Dental Sciences, Niigata, Japan; 6 Institute of Systems and Information Engineering, University of Tsukuba, Tsukuba, Japan; 7 Transdisciplinary Review Association, Moriya, Japan

**Keywords:** age-dependent effects, all-cause mortality, booster doses, COVID-19, mRNA vaccine

## Abstract

**Background/purpose:**

COVID-19 mRNA vaccines have been reported to reduce severe COVID-19 outcomes in high-risk populations. In Japan, booster vaccinations up to the seventh dose are publicly funded; however, evidence regarding the safety and potential benefits of repeated booster doses in younger adults remains limited. This exploratory observational study aimed to describe age-specific patterns of all-cause mortality according to the number of COVID-19 vaccine doses using municipal administrative registry data.

**Methods:**

Municipal registry data obtained through information disclosure requests from Hamamatsu City (2021–2024) and Matsudo City (2021–2025) were analyzed. The dataset included age group or year of birth (5-year increments), sex, date of residence, COVID-19 vaccination records (date and lot number), and date of death. Individuals aged 20–89 years were categorized into three age groups (20–49, 50–64, and 65–89 years). All-cause mortality rates were calculated per 100,000 person-years according to the number of vaccine doses. Exploratory comparisons between dose groups were conducted using Poisson tests. Because the registry dataset lacked information on comorbidities, healthcare utilization, socioeconomic status, and cause of death, multivariable adjustment and causal inference were not feasible.

**Results:**

Age-specific differences in all-cause mortality rates were observed across vaccine dose groups. Among individuals aged 65–89 years, higher numbers of vaccine doses were associated with lower all-cause mortality rates. In contrast, among those aged 20–49 years, all-cause mortality rates were higher among individuals who had received ≥5 doses than among those who had received fewer doses. Among individuals aged 50–64 years, the all-cause mortality rate was higher among those who had received six doses than among those who had received 1, 3, or 4 doses.

**Conclusions:**

This exploratory analysis identified age-specific differences in all-cause mortality across COVID-19 vaccine dose groups. However, the dataset available so far lacked important clinical covariates and information on causes of death, limiting causal interpretation. These findings highlight the need for constructing in Japan nationally linked datasets that allow adjustment for comorbidities, healthcare utilization, and other potential confounders.

## Introduction

Over 4 years have passed since COVID-19 mRNA vaccines were widely introduced in Japan, with their effectiveness and safety evaluated from various perspectives. Large-scale clinical trials have purportedly confirmed that these vaccines are highly effective in preventing severe disease and carry a low risk of serious adverse reactions. Initially, these were expected to serve as key tools for control (1).

Evidence from overseas studies has shown that a fourth booster dose reduced severe disease and hospitalization among high-risk groups such as older adults and immunocompromised individuals (2). Consequently, additional booster vaccinations are recommended at intervals of 6 months to 1 year. However, reports in Japan and abroad have described the risks of adverse events, including thrombosis, autoimmune disorders, and myocarditis (3).

In Japan, despite the relatively low number of COVID-19 cases and deaths in early 2020 compared with Western countries, the government pursued a nationwide vaccination campaign to achieve herd immunity. This has resulted in one of the highest per-capita vaccination rates worldwide (4). Booster doses were publicly funded for all residents, with recommendations extended to the seventh dose during the 2023–2024 winter season.

Nevertheless, since the emergence of the Omicron variant, both cases and deaths have increased in Japan. Reports indicate that excess mortality began to increase in 2021 and rose sharply in 2022 and 2023 (5), with approximately 100,000 excess deaths continuing into 2024. Multiple explanations have been proposed, including population aging, healthcare strain, delayed medical care, and indirect pandemic effects. Some have argued that vaccination itself may have contributed, although causality remains unproven (6, 7).

In Japan, the Vaccine Effectiveness Real-time Surveillance for SARS-CoV-2 (VERSUS) study by Nagasaki University found that additional doses greatly reduced severe illness but did not clearly prevent symptomatic infections (8). However, because the participants were limited to those seeking care, the study may be affected by care-seeking bias: vaccinated individuals are more likely to seek care for mild symptoms, whereas unvaccinated individuals may delay care until the illness is severe (9, 10). Since such differences can distort comparisons, test-negative designs like VERSUS should be interpreted with caution (11).

Currently, the magnitude of benefit of COVID-19 vaccination for younger adults remains uncertain. Questions remain regarding the value of frequent boosters in this group, considering waning effectiveness, cost-effectiveness, and the risk of adverse events (12). In Japan, annual boosters are recommended for older adults and individuals with comorbidities; however, younger adults and healthcare workers also receive boosters upon request. As younger adults are at a low risk of severe outcomes, evaluating potential risks, including adverse events and mortality linked to frequent boosters, is especially important.

Despite widespread vaccination, no domestic study has assessed the safety and mortality impacts of receiving frequent additional doses, stratified by age and the number of vaccinations. To address this gap, we obtained data linking resident registries and vaccination records from Hamamatsu City and Matsudo City in Japan through public information requests. We then integrated these datasets and conducted an exploratory analysis. The dataset included age group or year of birth (in 5-year increments), sex, date of residence, COVID-19 vaccination date (with lot number), and date of death. We analyzed three age groups−20–49, 50–64, and 65–89 years—focusing on the impact of frequent vaccination (≥5 doses) on all-cause mortality. This study aimed to explore possible associations between the number of vaccine doses and the all-cause mortality risk across age groups.

## Methods

This exploratory observational study used municipal administrative records. We analyzed resident data from Hamamatsu City in Shizuoka Prefecture (population approximately 780,000) and Matsudo City in Chiba Prefecture (population approximately 500,000). The datasets were obtained through public information requests submitted by NHK Fukuoka Broadcasting Station's “The Life” program. The data were provided to the authors on May 12, 2025. In each municipality, Basic Resident Register information (including birth, death, and relocation) was linked with COVID-19 vaccination records (including date, manufacturer, lot number, and number of doses) before the datasets were provided to the authors. No information that could identify individuals was included, as the datasets used in this study had been anonymized by the cities of Hamamatsu and Matsudo and no linkage tables were created. The datasets did not contain information on comorbidities, healthcare utilization, socioeconomic variables, or causes of death.

The Hamamatsu dataset covers the period between February 1, 2021 and June 30, 2024, and includes the following variables: age group (in 5-year intervals as of June 30, 2024), sex, vaccination history (dates, manufacturers, lot numbers), date of death, age at death (in 5-year intervals), date of becoming a resident, and date of leaving residency (due to death, relocation, etc.). If a resident moved in and out multiple times, only their first residency period was analyzed.

The Matsudo dataset includes residents as of February 1, 2021, with follow-up through March 31, 2025, and contains the following variables: year of birth (in 5-year intervals), sex, date of relocation (if applicable), date of death (if applicable), and vaccination information (dates, manufacturers, and lot numbers). To align with Hamamatsu's classification, Matsudo residents born between years *N* and *N*+4 were classified as aged (2021–*N*) through (2025–*N*), resulting in an approximately 1.5-year discrepancy compared to Hamamatsu's definition.

Vaccination status was treated as a time-updated exposure. No formal calendar-time adjustment was applied in the statistical analyses. The study population was stratified by the number of vaccine doses received at each observation point, and all-cause mortality rates were calculated using the person-years method. Person-years were accrued from cohort entry—or, for monthly all-cause mortality rates, from the beginning of each month—until death, relocation, or end of the follow-up period. For monthly all-cause mortality rates, person-years per individual were therefore capped at 1 month per observation period. Deaths were classified according to the most recently received vaccine dose. As individuals received additional doses, they contributed person-years sequentially to each corresponding dose category. For example, a person who received the first dose on April 1, the second dose on May 1, and the third dose on October 1 contributed person-time as follows: from study entry to April 1 in the 0-dose category; from April 1 to May 1 in the 1-dose category; from May 1 to October 1 in the 2-dose category; and after October 1 in the 3-dose category. If the individual died on December 1, the death was attributed to the 3-dose category.

Because vaccination records maintained by local governments are limited to records of emergency or routine publicly funded vaccinations, individuals with optional or temporary vaccinations were necessarily excluded from the study. The main observation period spanned from February 1, 2021, to March 31, 2024. The analyses covered the following: vaccination dose distribution by age group; all-cause mortality rates (per 100,000 person-years) by age and dose (10-year intervals) with and without a 90-day window after vaccination, with the latter further stratified into 20–49, 50–64, and 65–89 age brackets; death counts by days since the last vaccination (in 10-day intervals), and monthly all-cause mortality rates (per 100,000 person-years) by dose for each age group (to June 30, 2024); half-yearly death counts (July 2021 to June 2024) categorized by vaccination status (unvaccinated, 1–4 doses, 5–7 doses). Poisson tests were conducted between dose groups for simplicity, while accounting for multiple comparisons.

All analyses except for half-yearly death aggregations were performed using R (v4.5.1) with the IncidencePrevalence (v1.2.1), popEpi (v0.4.13), readxl (v1.4.5), scales (v1.4.0), and tidyverse (v2.0.0) packages. Half-yearly aggregations were performed in Python (v3.12), and the results were summarized in Microsoft Excel.

Because vaccination is a time-varying exposure, this simplified classification may introduce immortal time bias and survivorship bias. These limitations should be considered when interpreting dose-specific mortality patterns. More sophisticated time-dependent survival models were not feasible due to a lack of key covariates necessary for meaningful adjustment.

## Results

**Figure 1:**
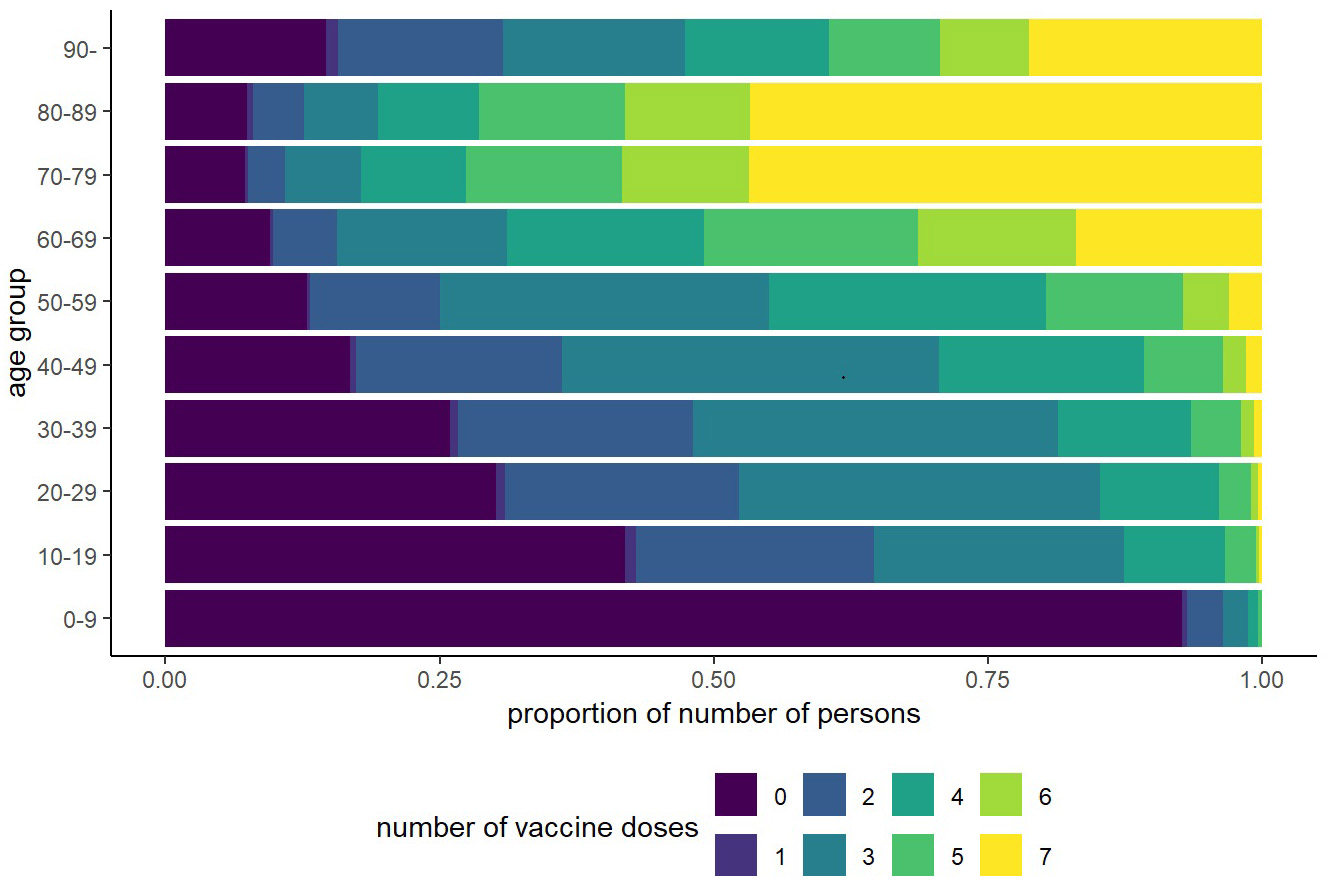
Distribution of the number of vaccine doses across age groups (expressed as proportions).

**Figure 2:**
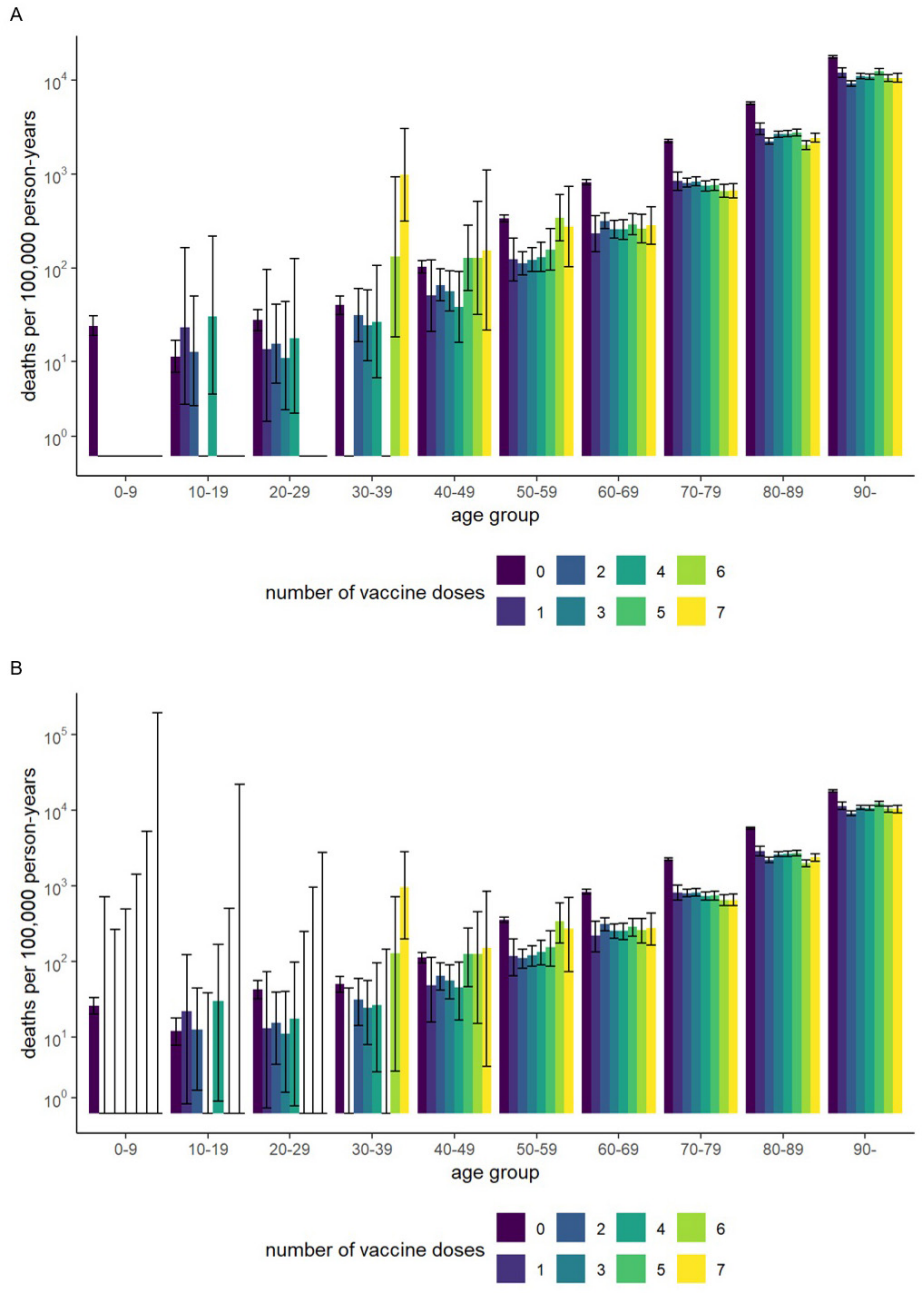
All-cause mortality rates (unstandardized) by age group and number of vaccine doses: **(A)** without a risk window, **(B)** with a 90-day window after vaccination. Error bars represent 95% confidence intervals.

Figure 1 shows the vaccination dose distribution by age, and Figure 2 shows the all-cause mortality rates by age and dose. Among individuals aged ≥60 years, all-cause mortality decreased with increasing doses. In the 20–49-year age group, all-cause mortality declined slightly up to four doses but increased among those who received five or more doses, although the confidence interval was wide in the younger age groups. This tendency persisted even when the 90-day window was applied to death counts after vaccination (Figure 2B).

**Figure 3:**
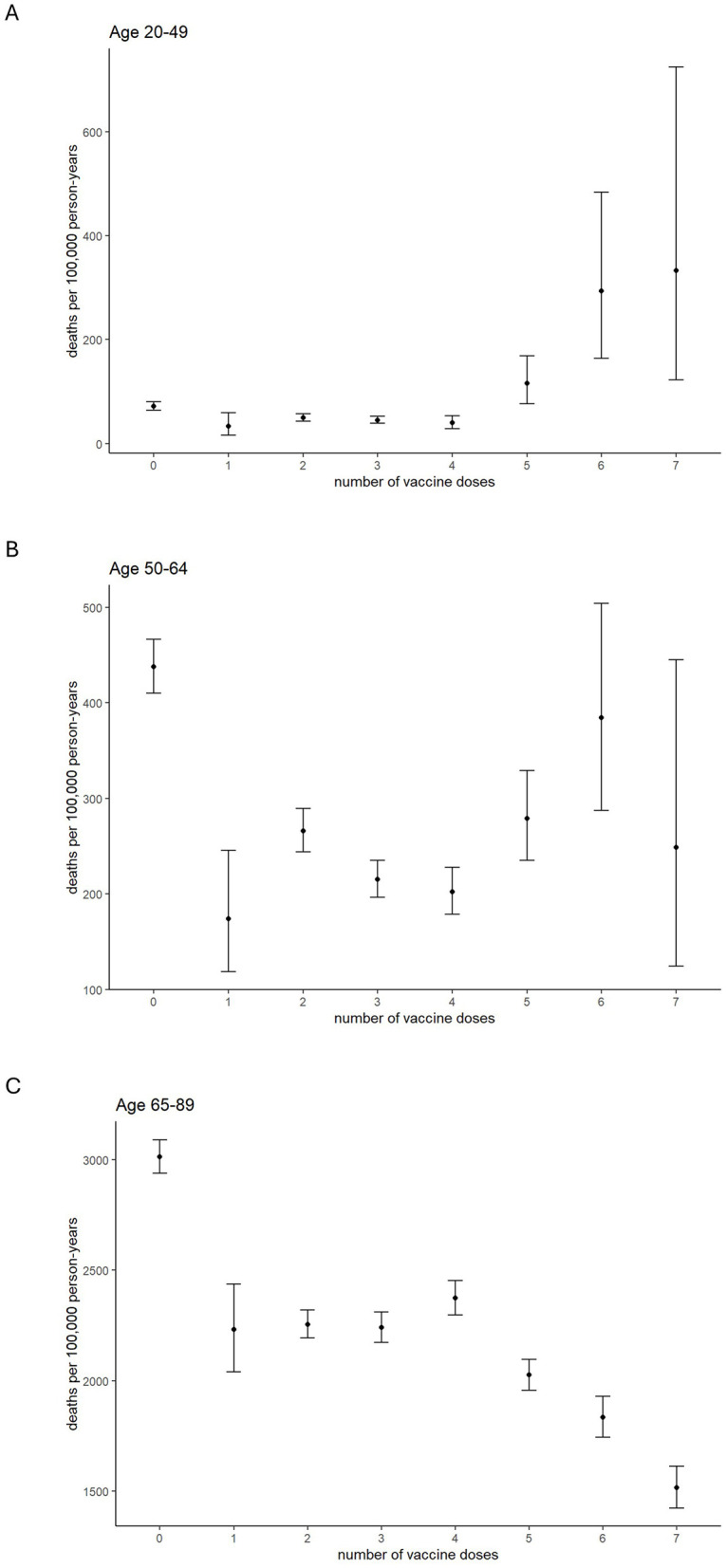
All-cause mortality rates (unstandardized) by number of vaccine doses. Ages 20–49 **(A)**, 50–64 **(B)**, and 65–89 **(C)**. Error bars indicate 95% confidence intervals.

Aggregated analyses (20–49, 50–64, and 65–89 years; Figure 3) showed similar patterns. Poisson's test results are presented in Supplementary Table 1. Among those aged 20–49, all-cause mortality rates were higher among individuals who had received ≥5 doses than among those who had received 1–4 doses, whereas in the 65–89 age group, all-cause mortality rates were lower in the higher-dose groups. In the 50–64 age group, the all-cause mortality rate was higher among individuals who had received six doses compared with those who had received 1, 3, or 4 doses.

**Figure 4:**
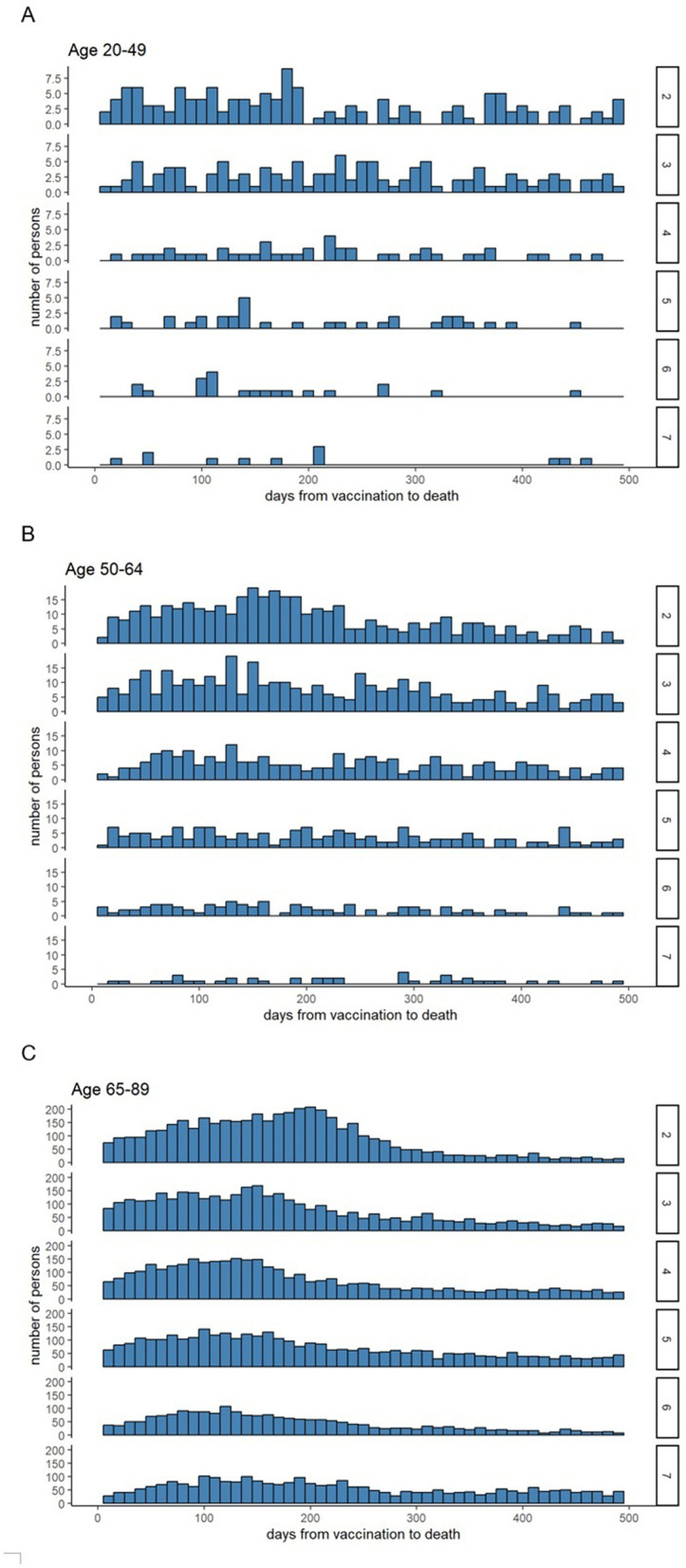
Distribution of days from vaccination to death. Ages 20–49 **(A)**, 50–64 **(B)**, and 65–89 **(C)**. The left axis displays the number of deaths in each 10-day interval, and the numbers on the right indicate the number of vaccine doses administered.

Figure 4 illustrates the interval between vaccination and death. In the 65–89 age group, deaths immediately after vaccination were rare, and all-cause mortality increased gradually before declining. For recipients of the six- and seven-dose vaccines, deaths immediately after vaccination were particularly low, followed by higher counts at later intervals.

**Figure 5:**
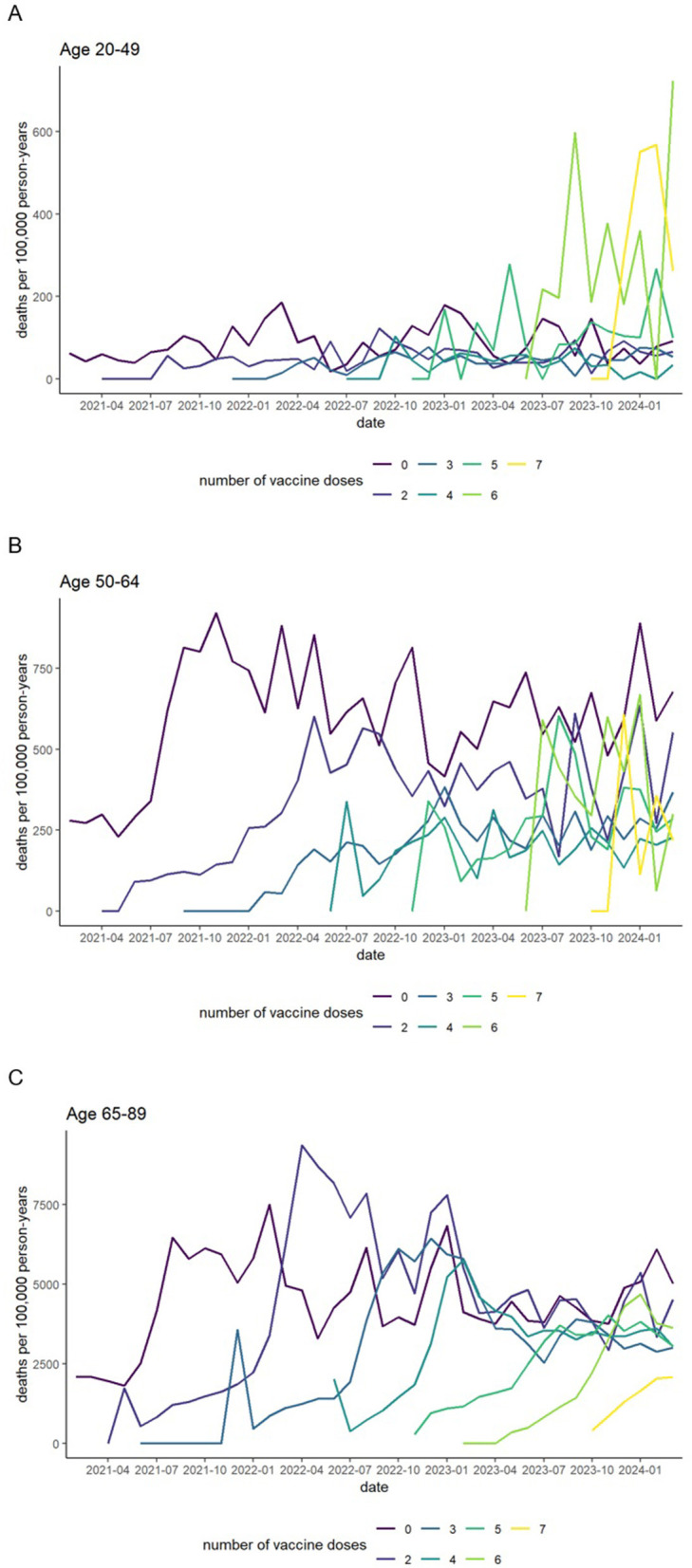
Monthly (calendar time) all-cause mortality rates (unstandardized) by number of vaccine doses. Ages 20–49 **(A)**, 50–64 **(B)**, and 65–89 **(C)**. Individuals who received only one dose were excluded due to their small number and the resulting large fluctuations in monthly mortality rates.

Figure 5 illustrates monthly all-cause mortality rates among individuals in each age group across the observation period. At each new vaccination round, all-cause mortality was low among newly vaccinated individuals but rose sharply among those who had received one fewer dose. No new rounds were initiated after the seventh dose, and no prior patterns were observed. In the 20–49 age group, all-cause mortality remained consistently low among individuals who received 2–4 doses throughout the observation period, whereas all-cause mortality was markedly higher among those who received 5–7 doses.

**Figure 6:**
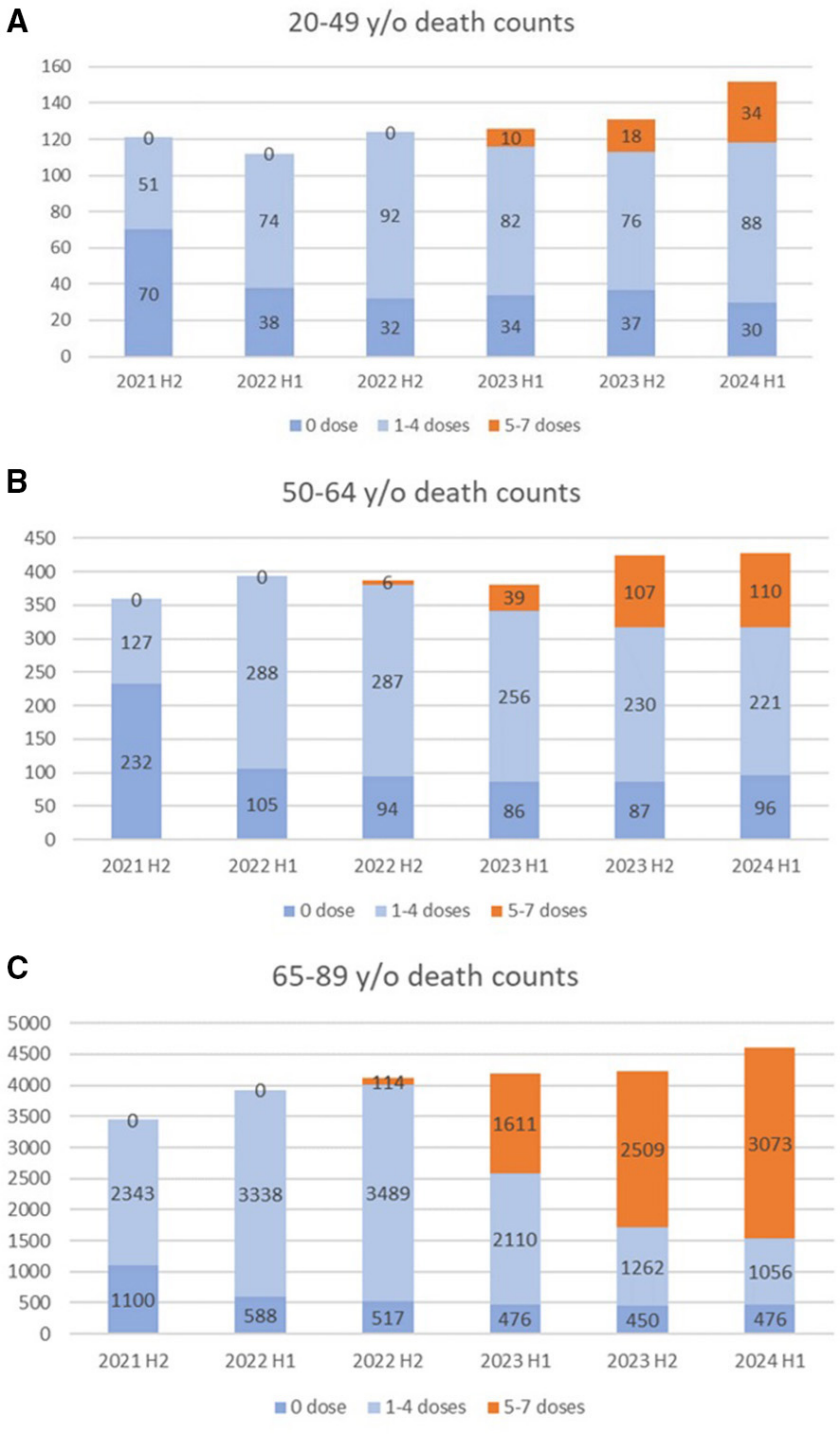
Time series of all-cause deaths among individuals with 0, 1–4, and 5–7 vaccine doses. Ages 20–49 **(A)**, 50–64 **(B)**, and 65–89 **(C)**.

Figure 6 presents the half-yearly death counts (July 2021–June 2024). In the 50–64 group, all-cause mortality declined in those with 1–4-doses, while all-cause mortality increased among those with ≥5-doses, particularly in late 2023. Similar trends were observed in the 65–89 group, with deaths among the ≥5-dose group rising in early 2024. Conversely, in the 20–49 group, all-cause mortality in the 1–4-dose group remained steady, while deaths in the ≥5-dose group increased markedly.

## Discussion

This exploratory analysis compared all-cause mortality across groups defined by the cumulative number of COVID-19 vaccine doses and identified age-specific differences in all-cause mortality patterns. Notably, among non-elderly individuals (20–49 and 50–64 years), a tendency toward higher all-cause mortality was observed in groups receiving more frequent vaccinations (≥5 doses).

At the same time, the overall body of international evidence generally supports the view that vaccination provides a net benefit in high-risk populations. Large-scale studies have demonstrated the safety and effectiveness of COVID-19 vaccines, particularly for the first one to three doses. Nationwide cohort studies conducted in several countries have not reported an increase in all-cause mortality among vaccinated individuals (13–15).

The situation examined in the present study—repeated vaccination (five to seven doses) within a specific subpopulation in Japan—differs substantially from the conditions evaluated in these large international studies, making direct comparisons difficult. The primary focus of the present study is therefore to provide information relevant to the safety of repeated booster vaccination among non-elderly populations.

### Sources of bias in observational vaccine studies

Observational studies of COVID-19 vaccine effectiveness are subject to several potential biases (16, 17). The high-risk-for-death effect, healthy vaccinee effect, and healthy user bias may reduce all-cause mortality in vaccinated groups by preferentially including healthier individuals, potentially overestimating vaccine effectiveness (18). Conversely, confounding by indication may lead to underestimation because vaccination is often prioritized for older adults and those with comorbidities who inherently have higher mortality risks. Differential depletion of susceptibility may also occur when high-risk individuals in the unvaccinated group die earlier, leaving a healthier survivor population. Frailty bias may further contribute to underestimation because frail individuals are frequently prioritized for vaccination.

Hamamatsu City, which contributed about half of the dataset, provides publicly available demographic information. The pre-pandemic all-cause mortality rate among individuals aged 65–89 years in 2019 was 2,456.2 per 100,000 (19), consistent with the all-cause mortality rate observed in the unvaccinated group in early 2021 (Figure 5). Although large differences in all-cause mortality were observed between unvaccinated and two-dose groups, COVID-19 deaths accounted for only 52 of 8,823 deaths (0.6%) in Hamamatsu City in 2021 (20), making it unlikely that COVID-19 mortality alone explains this difference.

After each vaccination round, a sharp increase in all-cause mortality was repeatedly observed in the N-1 dose group among individuals aged 65–89 years (Figure 5), whereas relatively few deaths occurred immediately after the sixth and seventh doses (Figure 4). This pattern suggests that individuals with poor health or prior adverse reactions were less likely to continue vaccination, leaving relatively healthier individuals in higher-dose groups. These findings indicate a potential healthy vaccinee bias, meaning the observed reduction in all-cause mortality may partly reflect selection of healthier individuals rather than biological protection alone.

This interpretation is consistent with a cohort study in Qatar reporting a pronounced healthy vaccinee effect during the first 6 months after vaccination, particularly among older and clinically vulnerable populations (21).

More reliable estimates would require adjustment for baseline health status and comorbidities using multivariable regression or propensity-score methods. In addition, attributing deaths to the last dose group poses methodological challenges for comparisons between dose categories. This approach should be re-examined if more detailed datasets can be obtained in future studies.

### Increased all-cause mortality with frequent vaccination in younger adults

The healthy vaccinee bias described above was also observed among younger adults, but it appeared to be limited to the early stages of the vaccination program. Its impact diminished after three doses in the 20–49 age group and after four doses in the 50–64 age group (Figures 5A, B). This pattern may partly reflect the lower vaccination coverage in younger populations and the sharp decline in the proportion receiving higher numbers of booster doses in these age groups.

A notable feature in the study population, particularly among individuals aged 20–49 years, was the elevated all-cause mortality rate among those who received five or more doses, with a similar pattern observed in the 50–64 age group, especially among those receiving six doses (Figure 3). Given Hamamatsu City's historical all-cause mortality rates in 2019−67.0 per 100,000 for ages 29–49 and 320.0 for ages 50–64 (19, 20)—the elevated all-cause mortality observed in this study warrants further investigation. One possible explanation is confounding by indication, whereby high-risk individuals are more likely to receive repeated vaccinations.

In Japan, COVID-19 booster vaccination campaigns were implemented according to priority groups defined by the Ministry of Health, Labour, and Welfare, as shown in Table 1. Booster vaccination prioritized healthcare workers, older adults, and individuals with underlying medical conditions, with the latter two groups prioritized because of their higher risk of severe disease and all-cause mortality. From the fourth dose onward, vaccination recommendations during the summer season were no longer directed at the entire population, but were primarily targeted at the aforementioned high-risk groups.

**Table 1:** Timeline of COVID-19 vaccination schedule and recommendations by the Ministry of Health, Labour, and Welfare in Japan.

17 Feb 2021	Priority vaccination for healthcare workers began ^*a*^
April 2021	Vaccination started for elderly people aged 65 and older ^*b*^
May 2021	Vaccination started for people under 65 with underlying conditions and residents of disability facilities ^*c*^
21 Jun 2021	Workplace vaccination program began ^*d*^
Dec 2021	Third dose began for healthcare workers (in principle, at least 8 months after the previous dose; targeted all residents aged 18 or older who had completed the initial vaccination series) ^*e*^
May 2022	Fourth dose began (for people aged 60 and older, high-risk individuals aged 18 and above, healthcare workers, and workers at elderly care facilities; interval of at least 5 months. The interval between the second and third doses was also shortened to at least 5 months) ^*f*^
20 Sep 2022	Fifth dose using the Omicron-adapted bivalent vaccine began (for individuals aged 12 or older who had completed the initial vaccination series and whose last dose was at least 5 months earlier) ^*g*^
21 Oct 2022	The vaccination interval for both the original Wuhan-strain vaccine and the Omicron-adapted bivalent vaccine was shortened to at least 3 months ^*h*^
8 May 2023	Sixth dose began (eligible groups: people aged 65 and older, individuals aged 5–64 with underlying conditions, and healthcare workers. The obligation to make efforts and active vaccination recommendation were applied only to people at high risk of severe illness, and healthcare workers were removed from the recommended group) ^*i*^
20 Sep 2023	Seventh dose began using the monovalent XBB.1.5-adapted vaccine. Eligible for individuals aged 6 months and older who had completed the primary vaccination series ^*j*^
31 Mar 2024	Special temporary vaccination program ended ^*k*^

^*a*^ https://www.mhlw.go.jp/content/000739551.pdf

^*b*^ https://www.mhlw.go.jp/content/000746894.pdf

^*c*^ https://www.mhlw.go.jp/content/000771359.pdf

^*d*^ https://www.mhlw.go.jp/content/000786973.pdf

^*e*^ https://www.mhlw.go.jp/content/000855973.pdf

^*f*^ https://www.mhlw.go.jp/content/000935002.pdf

^*g*^ https://www.mhlw.go.jp/content/000989882.pdf

^*h*^ https://www.mhlw.go.jp/content/001003599.pdf

^*i*^ https://www.mhlw.go.jp/content/001068073.pdf

^*j*^ https://www.mhlw.go.jp/content/001137294.pdf

^*k*^ https://www.mhlw.go.jp/content/001222703.pdf

Within the 20–49 age group, the proportion of individuals receiving five or more doses was much lower than in older groups. Therefore, the relatively high all-cause mortality observed among frequently vaccinated individuals may partly reflect a higher concentration of high-risk individuals in this group. A similar high-mortality trend was observed in the 50–64 age group among those receiving five to six doses (Figure 3).

As shown in Figure 6, the total number of deaths in the 0–4 dose group aged 50–64 declined gradually, while the increase in the 5–7 dose group kept the total death toll relatively constant. In contrast, deaths in the 0–4 dose group aged 20–49 remained stable even after the fifth round of vaccination, with the 5–7 dose group contributing to a surge in total deaths within this age group. If a considerable portion of high-risk individuals were more likely to receive the fifth and subsequent doses, all-cause mortality in the 0–4 dose group aged 20–49 would be expected to decline; however, this was not observed in our analysis. It is also noteworthy that no increase in all-cause mortality rate was observed in the 4-dose group in this age group (Figure 3A), even though the tendency not to receive a fourth dose is apparent in the younger subset of this group (Figure 1). If the higher all-cause mortality rate observed after the fifth and subsequent doses were primarily explained by confounding due to high-risk individuals being more likely to receive additional vaccinations, an increase in all-cause mortality might already be expected starting from the fourth dose. These findings suggest that selective vaccination of high-risk individuals may not fully account for the observed all-cause mortality rate differences, although residual confounding cannot be ruled out.

These patterns illustrate the difficulty of interpreting dose-specific mortality trends using observational registry data. The findings should therefore be interpreted as exploratory observations that generate hypotheses rather than evidence of causal relationships. Large-scale studies incorporating detailed clinical covariates will be necessary to clarify the potential benefits and risks of repeated COVID-19 vaccination.

### Policy context and evidence from other studies

Recent data from U.S. veterans indicate that the 2024–2025 COVID-19 vaccine reduced the risk of COVID-related emergency visits, hospitalization, and death over a 6-month period, with protective effects observed across age groups and comorbidity status (22).

As of October 2025, vaccination in Japan is primarily recommended for adults aged 65 years or older and individuals with comorbidities, although others may opt to receive it. Several Japanese academic societies continue to recommend vaccination for older adults because of their high risk of severe COVID-19 and the ongoing evolution of SARS-CoV-2 variants (23).

However, considering the all-cause mortality patterns observed in this study, the permissibility of unrestricted repeat vaccination among younger adults warrants further evaluation. Frequent vaccination was associated with lower all-cause mortality in older adults but higher all-cause mortality in younger adults, although these associations do not establish causality. The mechanisms involved—including the potential influence of vaccine type, dosing interval, and lot variation—remain unclear. These observations may represent potential pharmacovigilance signals requiring continued monitoring and further investigation.

### Implications for pharmacovigilance and data infrastructure

Several countries, including Taiwan, Denmark, and the United Kingdom, have established nationwide linked healthcare databases integrating vaccination records with healthcare utilization and mortality data (24–26). These systems enable large-scale pharmacoepidemiological analyses with adjustment for comorbidities and other confounders.

In Japan, however, vaccination records are primarily managed at the municipal level (27, 28), and linkage with national healthcare databases such as the National Database of Health Insurance Claims and Specific Health Checkups (NDB) remains limited for research purposes. Consequently, large-scale analyses using individual-level linked data are currently difficult to conduct.

The Japanese government is developing a national vaccine database designed to enable linkage with healthcare databases, including the NDB, under the Next-Generation Medical Infrastructure Development Act and the revised Vaccination Act (29). Once implemented, this infrastructure may allow more comprehensive pharmacoepidemiological analyses incorporating comorbidities, healthcare utilization, and cause-specific mortality.

## Strengths and limitations

The present study has several strengths. First, it uses complete administrative records with precise dates of vaccination and death. Second, it covers an extended observation period, including up to seven vaccine doses in certain populations. Third, the data are publicly available through an open repository, supporting transparency and reproducibility.

Several limitations should be considered when interpreting the findings. This exploratory observational analysis is hypothesis-generating, and the observed associations cannot be interpreted as causal. As in all observational studies, residual confounding and selection bias may produce spurious associations.

Methodological constraints further limit causal inference. Deaths were attributed to the most recent vaccination category, and the analysis did not use a formal time-to-event survival framework with time-varying exposure. Because vaccination status changes over time, failure to treat it as time-dependent may introduce biases such as immortal time bias and survivor bias. In addition, the dataset lacked key clinical covariates required for meaningful adjustment in time-to-event models, which limited the feasibility of more sophisticated survival analyses.

Confounding by indication is also a major concern. Individuals receiving repeated vaccinations may differ systematically from those receiving fewer doses with respect to baseline health status and medical risk. Therefore, it is not possible to disentangle the extent to which the observed excess mortality reflects higher baseline risk rather than any potential effect of vaccination.

Because individual-level data on comorbidities, healthcare utilization, lifestyle factors, and socioeconomic status were unavailable, these factors could not be adequately controlled, and the observed associations—particularly among those receiving five or more doses—may reflect underlying risk differences rather than vaccination effects.

The analysis was limited to all-cause mortality, as cause-specific mortality data were unavailable. Although all-cause mortality is an objective outcome, the absence of cause-specific analyses limits biological interpretation and prevents assessment of potential mechanisms.

Vaccination timing was also not modeled in relation to epidemiological context, such as epidemic waves, circulating variants, or policy changes, which may influence both vaccination behavior and mortality risk. In addition, individuals receiving five or more doses may accumulate more person-time in later phases of the pandemic, when background mortality levels may differ for reasons unrelated to vaccination.

Finally, the study population was limited to residents of two municipalities, Hamamatsu and Matsudo, which may limit generalizability to the broader Japanese population. Population movement during the study period was likely limited but could introduce minor bias.

The apparent age-dependent pattern—lower all-cause mortality among highly vaccinated older adults but higher all-cause mortality among highly vaccinated younger adults—should therefore be interpreted cautiously and may reflect differences in selection rather than biological effects. Without adjustment for baseline health status, comorbidities, and healthcare utilization, these age-specific associations cannot be clearly disentangled.

Overall, definitive conclusions regarding vaccine safety or effectiveness cannot be drawn, and the findings should be regarded as exploratory and requiring confirmation in larger datasets with more detailed clinical information.

## Conclusion

This exploratory study examined patterns of all-cause mortality across cumulative COVID-19 vaccination strata in two Japanese municipalities.

Analysis of resident data from Hamamatsu and Matsudo showed lower all-cause mortality among older adults (65–89 years) receiving ≥5 doses, likely reflecting healthy vaccinee bias rather than a direct vaccine effect. In contrast, all-cause mortality was higher among younger adults (20–49 years) with ≥5 doses and among those aged 50–64 with six doses. These findings suggest that the safety and effectiveness of repeated vaccination in non-elderly populations remain uncertain and that booster strategies for younger adults require careful pharmacoepidemiologic risk–benefit evaluation.

Although causality cannot be established because of limited covariates and potential residual confounding, the observed age- and dose-dependent mortality patterns highlight the need for constructing nationwide datasets adjusting for comorbidities, healthcare utilization, vaccine type, dosing interval, and cause-specific mortality.

## Data Availability

The datasets presented in this study can be found at https://github.com/covid-vaccine-jp/VRS.
