# Supplementary material for: Age-specific patterns of all-cause mortality across COVID-19 booster dose groups in two Japanese municipalities: an exploratory analysis
Source: Front Public Health. 2026 Apr 20;14:1795437. doi: 10.3389/fpubh.2026.1795437 (PMC13136084; doi:10.3389/fpubh.2026.1795437)
Supplement: Supplementary file 1 [file Table_1.pdf]

Supplementary Table 1. Results of Poisson tests for the data shown in Figure 3. The cells with $p < 0.05$ after Bonferroni correction are shown in bold.

		20-49 y/o		50-64 y/o		65-89 y/o	
dose	dose	p-value	**Bonferroni p**	p-value	**Bonferroni p**	p-value	**Bonferroni p**
0	1	8.51E-03	2.38E-01	4.15E-09	**1.16E-07**	2.60E-11	**7.29E-10**
0	2	5.65E-05	**1.58E-03**	1.17E-20	**3.27E-19**	3.22E-52	**9.02E-51**
0	3	2.07E-06	**5.81E-05**	8.05E-39	**2.25E-37**	2.21E-49	**6.19E-48**
0	4	9.39E-05	**2.63E-03**	1.90E-32	**5.33E-31**	2.63E-30	**7.35E-29**
0	5	2.48E-02	6.93E-01	1.47E-07	**4.12E-06**	4.02E-77	**1.12E-75**
0	6	9.51E-06	**2.66E-04**	4.19E-01	1.00E+00	1.36E-74	**3.81E-73**
0	7	2.28E-03	6.38E-02	6.36E-02	1.00E+00	2.70E-105	**7.55E-104**
1	2	2.40E-01	1.00E+00	1.84E-02	5.16E-01	8.51E-01	1.00E+00
1	3	4.12E-01	1.00E+00	2.79E-01	1.00E+00	9.62E-01	1.00E+00
1	4	7.49E-01	1.00E+00	4.80E-01	1.00E+00	2.07E-01	1.00E+00
1	5	3.73E-04	**1.05E-02**	1.29E-02	3.62E-01	4.58E-02	1.00E+00
1	6	1.38E-07	**3.87E-06**	3.60E-04	**1.01E-02**	1.74E-04	**4.87E-03**
1	7	1.41E-04	**3.96E-03**	3.32E-01	1.00E+00	5.68E-12	**1.59E-10**
2	3	4.31E-01	1.00E+00	7.61E-04	**2.13E-02**	7.60E-01	1.00E+00
2	4	1.89E-01	1.00E+00	2.25E-04	**6.31E-03**	1.90E-02	5.31E-01
2	5	1.74E-04	**4.88E-03**	5.99E-01	1.00E+00	1.79E-06	**5.01E-05**
2	6	1.25E-07	**3.49E-06**	1.36E-02	3.80E-01	5.15E-13	**1.44E-11**
2	7	3.56E-04	**9.97E-03**	1.00E+00	1.00E+00	6.42E-33	**1.80E-31**
3	4	4.66E-01	1.00E+00	4.28E-01	1.00E+00	1.12E-02	3.14E-01
3	5	4.48E-05	**1.25E-03**	7.46E-03	2.09E-01	1.77E-05	**4.97E-04**
3	6	4.49E-08	**1.26E-06**	2.35E-04	**6.57E-03**	1.04E-11	**2.91E-10**
3	7	2.28E-04	**6.39E-03**	6.21E-01	1.00E+00	1.53E-30	**4.28E-29**
4	5	2.96E-05	**8.29E-04**	2.04E-03	5.72E-02	5.25E-11	**1.47E-09**
4	6	2.86E-08	**8.00E-07**	6.57E-05	**1.84E-03**	7.12E-18	**1.99E-16**
4	7	1.45E-04	**4.06E-03**	4.94E-01	1.00E+00	5.99E-39	**1.68E-37**
5	6	7.31E-03	2.05E-01	5.24E-02	1.00E+00	1.26E-03	**3.52E-02**
5	7	2.82E-02	7.90E-01	8.81E-01	1.00E+00	2.25E-16	**6.30E-15**
6	7	8.05E-01	1.00E+00	2.41E-01	1.00E+00	2.36E-06	**6.61E-05**
